# Pharmacological targeting of cognitive impairment in depression: recent developments and challenges in human clinical research

**DOI:** 10.1038/s41398-022-02249-6

**Published:** 2022-11-17

**Authors:** Michael J. Colwell, Hosana Tagomori, Sarah Chapman, Amy L. Gillespie, Philip J. Cowen, Catherine J. Harmer, Susannah E. Murphy

**Affiliations:** 1 University Department of Psychiatry, University of Oxford, Warneford Hospital, Oxford, UK; 2 Oxford Health NHS Foundation Trust, Warneford Hospital, Oxford, UK

**Keywords:** Depression, Clinical pharmacology, Human behaviour, Molecular neuroscience

## Abstract

Impaired cognition is often overlooked in the clinical management of depression, despite its association with poor psychosocial functioning and reduced clinical engagement. There is an outstanding need for new treatments to address this unmet clinical need, highlighted by our consultations with individuals with lived experience of depression. Here we consider the evidence to support different pharmacological approaches for the treatment of impaired cognition in individuals with depression, including treatments that influence primary neurotransmission directly as well as novel targets such as neurosteroid modulation. We also consider potential methodological challenges in establishing a strong evidence base in this area, including the need to disentangle direct effects of treatment on cognition from more generalised symptomatic improvement and the identification of sensitive, reliable and objective measures of cognition.

## Introduction

Cognitive impairment is a core feature of major depressive disorder (MDD), broadly characterised by heterogeneous reductions in executive functioning, learning and memory [1–3]. Although cognitive impairment occurs in most clinical cases of depression (approximately 85–94%) [4], it is rarely a primary focus of routine clinical management compared with other core features of MDD (*e.g*. mood disturbances) [5–7]. Cognitive impairment presents inimical challenges to quality of life, is associated with poor functional outcomes [8–10], and is a key mediator of perceived disability in MDD [11, 12]. Consistent with this, our consultations with individuals with lived experience of depression highlight the impact of impaired cognition on daily living and service engagement (Box 1). Few treatment options for cognitive impairment in depression are available, with no recommended strategies in current clinical practice guidelines for MDD for the United Kingdom, United States or Germany [6, 13–16]. However, there are several promising novel pharmacological targets, with an increasing evidence base in humans.

In this review, we consider existing evidence from human clinical studies and evaluate potential new directions for pharmacological treatment development for cognitive impairment in MDD. We consider conventional agents which target primary neurotransmission, such as antidepressants, as well as novel pathophysiological targets. In addition, we highlight some methodological challenges of research in this area. The scope of the current review is limited to pharmacological approaches, although psychotherapeutic and transcranial electrotherapy stimulation approaches for cognitive impairment in psychiatric disorders are also being investigated [17–20].

The body of evidence in this narrative review was identified using a literature search to determine relevant human experimental studies, meta-analyses and systematic reviews (further details of the search strategy are described within these databases are described within Supplementary Material 2). Findings from the scientific literature were interpreted in the context of information gathered through consultations with individuals with lived experience of depression.

Box 1 Quality of life and clinical engagement – barriers arising from cognitive impairment identified by individuals with lived experience of depression During the development of the present review, we consulted with individuals with lived experience of depression to understand the impact of cognitive impairment on clinical recovery. Consultations were held with 13 individuals with lived experience of depression (ages 20–55; 12 females, 1 male; mixed ethnic backgrounds), in group meetings and one-to-one sessions with research team members. Consultations followed a semi-structured format (see online Supplementary Material 1), with questions related to experiences of cognitive difficulties associated with depression.

Group members shared experiences of diminished quality of life related to cognitive difficulties experienced during depression. Cognitive symptoms were associated with difficulties in engagement with a range of day-to-day activities, as well as clinical support:

- “*I dropped out of college because I couldn’t concentrate. At that point, my depression was at its worst, [I] dropped out, and couldn’t function*.”
- “*When I started to become depressed, I noticed I wasn’t able to concentrate on what people were saying. I found it very difficult to cope*.”
- “*It impacted my ability to organise appointments and meetings – because I’ve missed many of these, there have been a lot of times I’ve missed out on support*.”
- “*It was hard to remember the strategies and exercises learned*.”

Group members also shared difficulties with communication during depressive episodes, for example when interacting with clinicians:

- “*It was really difficult to describe what had happened [in the last week] and how things had built up when you just don’t have the words*.”
- *“Often talking to people felt overwhelming, and communicating with doctors felt impossible, meaning often it wasn’t possible to get any help.”*

***Note****: quotations are transcribed ad verbatim from consultations*.

## Pharmacological agents targeting primary neurotransmission

### SSRIs and SNRIs

Systematic reviews and meta-analyses of the effects of conventional antidepressant treatment (compared with placebo) on cognitive function in MDD have reported modest positive effect sizes, including improvements across domains of psychomotor speed and delayed recall [21–23]. However, these analyses pool data from a broad range of pharmacological agents, including the multimodal agent vortioxetine and the cholinergic agent donepezil [24, 25], which may have inflated the effects seen. Indeed, in one meta-analysis, the effect of antidepressant treatment on cognition became non-significant when vortioxetine studies were excluded [26]. Further, these meta-analyses included heterogenous clinical populations (e.g. depression in Parkinson’s disease), and many studies did not use standardised cognitive assessment batteries, instead making use of highly variable cognitive tasks which lack specificity for cognitive domain measured.

More direct evidence from a parallel-cohort randomised clinical trial (RCT) showed 8-weeks of standard antidepressant monotherapy with sertraline, venlafaxine, or escitalopram had no effect on cognitive performance in MDD across standardised assessments [27]. This evidence is particularly compelling given the large sample size (*n* = 1008), prospective design (with both pre- and post-treatment assessments) and healthy volunteer control group (to control for non-specific effects of repeated testing and symptomatic changes). These findings are consistent with accounts from our consultations with individuals with lived experience of depression, who often reported that antidepressants did not improve their cognitive impairment (Box 2).

Measuring the direct cognitive effects of drugs which primarily affect mood symptoms of depression is challenging. As depression remits, self-report (subjective) and objective cognitive impairments may not improve in parallel [28, 29]. Further, as subjective cognitive impairment is a symptom of depression within ICD-10 [30] and DSM-V [31] diagnostic classifications, and is measured on most standardised outcome measures for MDD [32–34], its improvement may contribute to indices of treatment response/remission. Antidepressants may also change non-specific factors such as motivation, which may increase effortful performance in cognitive tasks leading to improvements in task performance that do not reflect genuine gains in cognitive functioning – a methodological limitation known as pseudo-specificity [35, 36]. Such effects are infrequently controlled for in investigations of the cognitive effects of antidepressants.

Box 2 Experience with services and lack of effectiveness of standard treatment on cognitive impairment – accounts from individuals with lived experience of depression According to our lived experience consulatations, cognitive impairment was not discussed or considered by clinicians such as psychiatrists or clinical psychologists during engagement with services. Group members frequently highlighted that their treatment (*e.g*. SSRIs and psychotherapy) did not help with the cognitive difficulties they experienced.

- *“My psychiatrist didn’t mention cognitive impairment; I wish they had as I would have been able to put coping strategies in place and know that I’m not failing, I’m just not functioning properly.”*
- *“SSRIs didn’t help with memory and concentration.”*
- *“It would have been useful if my issues with concentration could have been addressed with standard treatment.”*

### 5-HTR modulators

Selective targeting of serotonin receptor subtypes may hold potential to more directly modify cognitive functioning [37]. Vortioxetine, a multimodal SSRI with significant affinity for various 5-HT receptor subtypes is the only agent recognised by the FDA as indicated for cognitive impairment in MDD [38]. In particular, vortioxetine is a potent antagonist of the 5-HT_3A_ receptor where it has a 10–38-fold greater affinity compared to the other 5-HT receptor subtypes at which it is active (5-HT_1A,_ 5-HT_1D,_ and 5-HT_7_) [39]; these broad serotonergic effects are thought to result in downstream modulation of glutamatergic signalling [24, 31, 40]. In multiple placebo-controlled RCTs, eight-weeks of vortioxetine monotherapy in MDD improved domains of executive functioning, learning and memory, with particularly well-replicated improvements on the Digit Symbol Substitution Test (DSST) [41–43]; the DSST is a highly sensitive measure of cognitive impairment with low cognitive domain specificity [44]. In a study of healthy volunteers (*n* = 48) and remitted MDD (*n* = 48) [45], vortioxetine but not placebo improved executive functioning performance; in addition, greater prefrontal and hippocampal activation was observed during a working memory task following vortioxetine administration, although no effect on objective memory performance was found. In contrast, a healthy volunteer study (*n* = 24) found no increase in cognitive functioning following vortioxetine administration [46]. Although the beneficial effects of vortioxetine on cognition in MDD are well-replicated, further work is needed to further understand the inconsistent cognitive effects observed in healthy volunteers.

Through path analysis of three clinical studies, McIntyre et al. [47] found DSST improvements following vortioxetine were independent of overall symptomatic improvement on the Montgomery-Asberg Depression Rating Scale (MADRS) [48]. However, it is important to note that these analyses of overall MADRS score do not exclude the possibility that specific symptoms mediated the effects on cognition [49]. For example, anhedonia may decrease motivation and effortful performance in executive functioning tasks [50, 51], and vortioxetine may be efficacious in remediating anhedonic dimensions of MDD [52]; by comparison, other symptom dimensions within the MADRS, such as reduced appetite, are less likely to mediate improved cognition.

Beyond vortioxetine, there is emerging evidence that selective agonists of serotonin receptor subtypes may hold promise as pro-cognitive treatments. For example, an open-label RCT (*N* = 89), the partial 5-HT_1A_ agonist buspirone, administered as an adjunct to escitalopram for eight weeks, demonstrated improved working memory in MDD compared with escitalopram monotherapy [53]. In contrast, a single dose of buspirone did not affect cognition in healthy volunteers [54]. Tandospirone, a structural analog of buspirone which selectively targets 5-HT_1A_ receptors with high agonist efficacy [55], similarly improved cognitive functioning in older adults with vascular dementia and anxiety (*N* = 89) [56], when given as an adjunct to escitalopram for eight weeks, compared with escitalopram monotherapy. In contrast, a single dose of tandospirone has been found to dose dependently impair explicit verbal memory in a small sample (N = 9, crossover design) of healthy male volunteers [57]. These limited studies show promise for 5-HT_1A_ as a target for cognitive impairment in depression, although its paradoxical effects in healthy volunteers require further elucidation.

In summary, there is a body of support for the idea that selective 5-HT receptor agonism may be a useful target for cognitive impairment in depression. However, it is important to note that many current 5-HTR modulator agents, such as buspirone and vortioxetine have complex neuropharmacological actions and may also impact cognition via other mechanisms, such as direct pro-dopaminergic [58] or indirect glutaminergic modulation. Further investigation of more selective agonists of 5-HTR subtypes, such as the 5-HT_4_ receptor agonist prucalopride [59], will further elucidate potential serotonergic targets for cognitive amelioration in MDD. Additionally, many novel 5-HT agonists have recently, or are currently, crossing the clinical threshold, including 5-HT_6_ receptor agonists and 5-HT_7_ receptor antagonists [60, 61]. These agents appear to improve cognition in both healthy and neurological rodent models (including Alzheimer’s disease and schizophrenia) [62–71], and serve as promising novel targets for ameliorating cognitive impairment in MDD.

### Dopaminergic modulators

A large body of evidence supports the regulatory role of dopaminergic signalling in cognitive functioning [72–74]. Consistent with this, pharmacological manipulation of dopaminergic signalling function with piribedil (D_2_ and D_3_ receptor agonist) and methylphenidate (inhibitor of dopamine transporters [DAT]) results in well-replicated improvements in cognitive performance in healthy individuals [75–77]. It is therefore interesting to consider whether dopaminergic antidepressant agents, such as bupropion, might have the potential as treatments for cognitive impairment in MDD.

Bupropion (which inhibits the reuptake of dopamine and noradrenaline) has been shown to improve cognitive function in MDD when taken as an adjunct to other antidepressants or as monotherapy [78, 79]. For example, improved visual and verbal memory and executive functioning was observed in patients with MDD after 8-week administration (*N* = 36) [79], although this effect was not apparent in a separate study of healthy volunteers [80].

Modafinil (which has a complex mechanism of action, including weak inhibition of dopamine reuptake) has also been shown to have pro-cognitive effects in patients with depression. In currently depressed patients 4-weeks administration of modafinil improved executive function (*N* = 31) [81]. In patients who have recovered from depression, modafinil was shown to improve episodic memory but not executive functioning after one week in remitted depression (*N* = 60) [82]. This evidence is consistent with multiple studies in healthy adults demonstrating broad improvements in verbal and visuospatial working memory, learning, attention and executive functioning following modafinil administration [81, 83–86].

Although modafinil and bupropion overlap mechanistically as inhibitors of DAT, bupropion acts on ≤22% of DAT binding sites [87], while modafinil produces weak atypical inhibition of DAT [88, 89]. Bupropion also blocks the reuptake of noradrenaline, with downstream modulation of tumour necrosis factor alpha and upregulation of brain-derived neurotrophic factor (BDNF) in MDD, which may be an alternative intra- and extracellular mechanism by which it exerts its antidepressant and cognitive effects [90, 91]. Similarly, the cognitive effects of modafinil may be explained by intracellular actions, including decreased neuronal free radicals, adenosine 5′-triphosphate production, and promotion of cellular metabolism [89]. Further, given the role of the mesolimbic-dopamine circuitry in reward processing and motivation, it is important to consider the extent to which the cognitive effects of bupropion and modafinil are related to non-specific changes in motivation and affect [35, 36].

### NMDA antagonists, AMPAkines, and metabotropic glutamate receptor inhibitors

Glutamatergic neurotransmission accounts for most excitatory activity in cortical structures, and is a predominant regulator of cognitive and sensory functioning [92, 93]. Glutamatergic transmission has gained much attention within the context of depression following the discovery of the potent antidepressant effects of the N-methyl-d-aspartate (NMDA) antagonist ketamine [94]. A single subanaesthetic infusion of ketamine has rapid, transient antidepressant effects in treatment-resistant depression (TRD) [95, 96]. Interestingly, several TRD studies have reported improved cognitive function postinfusion, including improved executive function, visual memory and complex working memory [97–99]. Ketamine is also associated with a reduction in suicidal ideation and planning, which it has been suggested could result from improved inhibitory control [100, 101]. The cognitive effects of ketamine may be attributable to the rapid promotion of neuronal plasticity via intracellular protein modulation, including rapamycin complex 1 and BDNF [102, 103], and inhibition of excitotoxicity through modulation of ionotropic and metabotropic glutamate receptors (mGluRs) [104].

It is unclear if ketamine has a direct effect on cognitive function, or if its cognitive effects are an indirect result of its rapid antidepressant properties. In two active placebo-controlled RCTs by Shiroma et al. [105] (*N* = 43) and Murrough et al. [106] (*N* = 62), postketamine-infusion improvements in speed of processing and working memory were independent of antidepressant response; however, the cognitive change reported in Murrough et al. [106] was associated with a significant main effect of time only and there were no differences in cognition between the ketamine and active placebo, suggesting the cognitive improvement may have been driven by a non-specific learning effect. An RCT of similar design by Liu et al. [107] (*N* = 50) found that change in speed of processing post-ketamine was associated with improved anxiety symptoms comorbid to TRD, and improved visual learning and memory performance were associated with improved depressive symptoms in TRD without comorbid anxiety. Two further studies investigating the cognitive effects of ketamine found a moderating effect of depressive symptom improvement on DSST performance and self-reported cognitive deficits, but not visual attention and task-switching performance [108], and a relationship between depressive symptomatic improvement and response inhibition performance [109].

Paradoxically, acute ketamine may induce cognitive impairment in some circumstances: an RCT of non-refractory MDD showed a single subanaesthetic dose reduced performance in executive function, attention and verbal memory [110]. Healthy volunteer studies have demonstrated similar reductions in episodic and working memory, attention and long-term memory both during and 1-hr post-infusion [111–114], with a return to baseline functioning 3 days post-infusion [115]. Interestingly, fronto-striatal functional connectivity increases in individuals with TRD and decreases in healthy individuals 2-days postinfusion [116], mirroring the opposing effects of ketamine on cognitive ability across these populations. Taken together, the apparent beneficial effects of ketamine on cognition may be specific to TRD, although further work is required. Additionally, the cognitive effects of ketamine may vary depending on dose frequency (single dose vs. repeat dose) and length of treatment [97].

Beyond ketamine, other glutamatergic agents have been investigated for potential cognition improving effects in MDD, including other NMDA antagonists, glutamatergic inhibitors, positive allosteric modulators of AMPAR (AMPAkines) and modulators of mGluRs. The NMDA antagonist riluzole has well-replicated antidepressant effects [117, 118], but did not improve self-reported cognitive impairment when administered as an adjunct to antidepressants [118]. Another NMDA receptor antagonist, memantine, has been shown to have antidepressant effects when administered as an adjunct to escitalopram [119–121]. Compared with escitalopram monotherapy, memantine and escitalopram combination therapy improved verbal memory and executive functioning in older adults with MDD [119] (*N* = 62), although not in younger adults with MDD (*N* = 80) [122]. The AMPAkine, Org 26576, improved executive functioning and working memory at high doses (*n* = 10) compared with low dose (*n* = 10) and placebo (*n* = 10) in MDD [123]; however, replication in a large cohort study is required [124]. In healthy older adults, an acute dose of a similar AMPAkine (Org 24448) improved short-term memory and executive functioning, but impaired episodic memory [125].

Excitotoxicity and consequential oxidative stress via NMDA hyperactivity is posited as a pathophysiological basis of cognitive impairment in MDD [126, 127], with group I and II mGluRs potentially inhibiting these effects [128, 129]. Although a developing research area, the first mGlu_2/3_R modulator (decoglurant) to advance to clinical study did not affect objective cognition nor depressive symptoms in an RCT of partial-refractory MDD (*N* = 357) [129, 130].

### Cholinesterase inhibitors

Cholinergic signalling plays a key role in memory processing and cognitive decline [131, 132]. Meta-analyses have demonstrated that cholinergic agents such as citicoline (cholinergic donor for acetylcholine synthesis [133]) administered in neurological populations (*e.g*. ischemic stroke) facilitate functional and cognitive recovery [134, 135], although these effects are not always replicated [136, 137].

Of the cholinesterase inhibitors investigated within the context of depression [138], donepezil is among the most promising and frequently studied [139]. A two-year placebo-controlled RCT investigating donepezil administered as an adjunct to antidepressants (escitalopram or duloxetine) reported improved global cognition (visuospatial functioning, language processing, executive functioning, delayed memory and processing speed) after one year in older adults with remitted MDD (*N* = 130); however, these effects did not persist at year two [140]. Similarly, in a pilot placebo-controlled RCT, donepezil administered as an adjunct to open-label antidepressant therapy improved verbal episodic memory in older adults with MDD (*N* = 12), although there was no effect on executive functioning or attention [141]. In contrast, another placebo-controlled RCT [142] showed that 16-weeks of donepezil administered as an adjunct to citalopram or venlafaxine did not improve cognitive function. These conflicting results may possibly be explained by the measures of cognition used in each study; cognitive improvements were observed following donepezil pharmacotherapy (*i.e*. [140, 141]) in studies in which batteries of standardised neuropsychological tests not specific to neurological disease were employed (e.g. WAIS-R). In contrast, studies in which no effects were observed (*i.e*. [142]) used the Alzheimer’s Disease Assessment Scale-Cognitive Subscale [143], which may not be sufficiently sensitive to changes in cognitive function in depressed patients and healthy volunteers [144].

Although donepezil appears promising as a potential cognitive ameliorating agent for MDD, it is important to note that no studies have yet investigated the effects of donepezil on cognitive functioning in younger adults with MDD. Moreover, the cognitive profile of donepezil in healthy volunteer studies is inconclusive; when acutely administered in healthy adults, donepezil improved cognitive function in two studies [145, 146], while it impaired cognition in two further studies [147, 148].

Administration of galantamine, another cholinesterase inhibitor, as an adjunct to antidepressants was shown to have no effect on cognitive function or mood symptoms in older adults with MDD (*N* = 38) in a placebo-controlled RCT [149]. Consistent with this, galantamine monotherapy did not change cognitive function or mood symptoms in adults with partially remitted MDD in another placebo-controlled RCT [143], although this study had a small sample size (*N* = 19).

Although galantamine and donepezil are both cholinesterase inhibitors [150], they have divergent secondary mechanisms of action which may explain differences in their cognitive profiles; in particular, donepezil but not galantamine is a potent agonist of sigma-1 receptors (σ1R) [151]. σ1R agonism is associated with promotion of neuroplastic and neuroprotective processes [152, 153], in addition to amplifying signal transduction across glutaminergic and dopaminergic pathways [143, 154, 155].

## Beyond primary neurotransmission – novel clinical targets

Recently, novel approaches to the pharmacological targeting of cognitive impairment have emerged. The proposition that cognitive impairment in MDD may be the result of progressive neurotoxic and neuroinflammatory processes, as well as volumetric reductions in neuroanatomical areas such as the hippocampus, striatum, and fronto-cingulate cortices [126, 156–161], suggests the modulation of neurosteroid, neurotrophin and pro-inflammatory cytokine activity might be useful targets for treatment development [126, 162–165].

Neurosteroid dysregulation is suggested to play a role in the pathophysiology of stress, neuroinflammation, and depression [165–170]; thus pharmacologically targeting neurosteroid dysregulation may be a useful approach for the treatment of depression and cognitive impairment. In particular, excitatory neurosteroids such as dehydroepiandrosterone and pregnenolone sulphate modulate the function of glutamatergic signalling pathways, thus promoting long-term potentiation [171–173]. Consistent with this idea, dehydroepiandrosterone administration was shown to improve verbal memory function in older adults with MDD in a small (*N* = 6) proof-of-concept study [174, 175]. Two Cochrane systematic reviews concluded there is inadequate evidence supporting a positive effect of dehydroepiandrosterone on cognitive function in healthy older adults and those presenting with age-related cognitive decline [176, 177]. However, a more recent small-scale placebo-controlled study in healthy young males (*N* = 24) showed dehydroepiandrosterone administration improved verbal episodic memory via cortisol inhibition [178].

Modulation of the σ1R ligands is another potential target for the treatment of cognitive impairment in MDD [179]. Fluvoxamine, an SSRI with high-affinity σ1R agonist properties [180], has been shown to be associated with improvements in the Wechsler Adult Intelligence Scale (WAIS-R) and DSST. However, the effects of fluvoxamine on cognition have not yet been tested against a placebo control; in a double-blind RCT [182], the effects of fluvoxamine were compared with the tetracyclic antidepressant mianserin which is known to have cognition-impairing effects [183]. In another, [184], cognitive improvements were observed only in treatment responders, suggesting a potential confounding effect of symptomatic improvement.

The cognitive effects of σ1R agonists such as fluvoxamine and donepezil are difficult to separate from their serotonergic and cholinergic properties, respectively. Studies using comparator agents such as sertraline and galantamine may further elucidate these effects [179], controlling for potential affective changes associated with neurosteroid modulation [185].

Stimulation of mineralocorticoid receptors via fludrocortisone may indirectly influence cognition [186]. In healthy volunteers (*n* = 24) and depressed adults (*n* = 24), verbal memory and executive functioning improved after fludrocortisone administration, with improved verbal memory associated with cortisol inhibition [187]. However, in older adults with MDD (*N* = 23), a similar experimental paradigm resulted in impairments of psychomotor speed, verbal learning and memory, and executive functioning [188].

Melatonin, when administered as an adjunct to buspirone, was associated with decreased self-reported cognitive impairment in antidepressant non-responders, indicating a potential pro-cognitive effect that is independent of pseudo-specific effects (*N* = 113) [189]. In multiple studies of healthy individuals, melatonin improved cognitive ability [190], although it is unclear if these cognitive gains might be due to improved sleep.

Erythropoietin improved verbal memory compared with placebo in individuals with TRD following 8 weeks administration (*N* = 40) [191]. In an additional study, individuals with MDD (*n* = 36) showed broad improvements in cognitive functioning (including memory, learning and executive functioning) and self-reports of cognitive function following 8 weeks of erythropoietin versus placebo [192]. In healthy volunteers, erythropoietin produced broad enhancements in executive function, memory and hippocampal long-term potentiation [193–195]. While promising, the exact mechanisms for cognitive change remain unclear; it has been posited these findings are a result of upregulated stromal cell-derived factor 1 and BDNF [126, 196], however, down-regulation of plasma BDNF following 8 weeks of erythropoietin administration in TRD has been reported independently [197].

The methyl donor, *S*-Adenosylmethionine, given as monotherapy for MDD resulted in similar depressive symptom reductions to escitalopram in two double-blind RCTs (*N* = 130; *N* = 189) [198, 199]. Antidepressant non-responders (*N* = 46) showed improved self-report of cognitive function following 6 weeks of adjunct S-Adenosylmethionine treatment [200, 201]. Recent metabolomic investigations highlighted the potential of exogenous *S*-Adenosylmethionine to upregulate toxic metabolite adenine, hindering the clinical practicality of this agent [202].

Taken together, although many of the novel pharmacotherapeutic targets of cognitive impairment in MDD reviewed here appear promising, vigorous independent replication is required to fully elucidate their clinical potential.

## Methodological challenges

In reviewing this literature, numerous methodological challenges are apparent in establishing a strong evidence base to support pharmacological targets for the treatment of cognitive impairment in depression.

### Pseudo-specificity

A fundamental challenge in establishing evidence of therapeutic validity for drugs which target cognitive function is to identify effects that are primarily mediated by changes in affect (pseudo-specificity). McIntyre et al. [203] posits this can be achieved where depressive symptomatology is appropriately adjusted for, using path analysis and subgroup analysis. Many RCTs have since employed this approach (e.g., [47, 105, 106, 204]), although much of the literature reviewed here has not adopted this methodology. It is also important to consider the influence of other affective domains beyond core depression symptoms, such as motivation, alertness/fatigue, and anxiety, as these may all have an indirect effect on cognitive task performance [205–209].

### Inconsistency of cognitive effects across clinical and non-clinical populations

A phenomenon commonly emerging in the reviewed literature is that pharmacological agents often lack shared and domain-specific cognitive effects between individuals with MDD and healthy controls. For example, buspirone and bupropion have both been shown to have cognitive-enhancing effects in patients with MDD [53, 78] but not in healthy volunteers [54, 79, 80]. In contrast, modafinil and citicoline broadly improved cognition in neurological and nonclinical populations, but showed limited or no change in MDD [81, 89, 210]. Furthermore, fludrocortisone resulted in cognitive improvement young adults with MDD but impaired cognitive function in older adults with MDD [187, 188].

One explanation for these differences between patient and healthy control studies is that pharmacologically-induced improvements in cognition may only be seen in those with cognition-related pathophysiological abnormalities, such as progressive neurotoxicity and reduced neurogenesis [126, 211]. For example, reduced grey and white matter integrity in frontal-limbic networks are both associated with cognitive impairment in MDD [212–215] and reduced neurotrophin and proinflammatory cytokine activity in MDD [216–219]. Indeed, baseline BDNF, including mature BDNF, and pro-inflammatory cytokine levels in individuals with MDD predicts cognitive improvement and antidepressant effects across a range of agents, including sertraline and vortioxetine [220–223], although it is important to note that peripheral measures of BDNF may not reflect central BDNF concentration [224]. Further, age and adjunctive therapy may influence antidepressant pharmacodynamics, where the former might explain the inefficacy of cholinergic agents for cognitive impairment in young adults compared with older adults [225]. To better understand pharmacologically-induced cognitive effects across specific populations, the identification of shared and independent treatment-response biomarkers would be beneficial [226, 227]. In particular, if early treatment-induced changes in such biomarkers (e.g. changes in the function of relevant neurocircuitry) were predictive of subsequent efficacy in treating cognitive symptoms, they would lend valuable support to drug development decision-making in this field.

An alternative explanation for inconsistent effects across healthy volunteer and patient samples is that the cognitive tasks used in many studies have insufficient sensitivity to detect pharmacologically-induced cognitive changes in healthy volunteers. Healthy volunteer studies often include high-functioning individuals who perform at ceiling on many standard neuropsychological tasks, thus limiting the detection of pharmacologically induced changes [228, 229]. The use of implicit, automatic measures of cognition may be potentially useful for increasing the sensitivity of pharmacologically-induced cognitive effects in healthy volunteers [77, 228].

### Heterogeneity of cognitive impairment in MDD

**Fig. 1:**
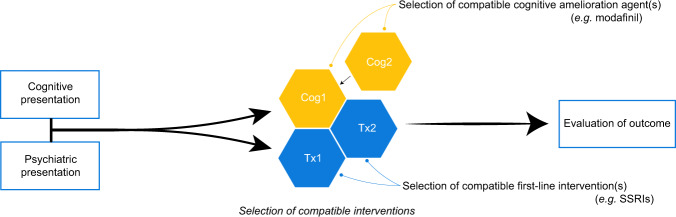
Modular considerations for managing depressive symptoms and cognitive impairment. Through assessment of both cognitive and psychiatric presentation, future research may provide scope for the identification of appropriate polypharmacy for the individual case.

Cognitive impairment in depression is heterogeneous in presentation, due to the pathophysiology of depression itself and external factors such as effects of medication [230]; impairments in specific domains of cognitive function such as memory, executive function and learning differ case-by-case [231, 232]. In future, a better understanding of the specific effects of pharmacological agents on different cognitive domains, when given alone or as an adjunct to antidepressant treatment, may facilitate the more targeted and personalised treatment of cognitive impairment in depression (Fig. 1). It will also be important to define the conditions necessary for successful clinical use of these agents, and where their clinical practicality lies; in particular, whether these agents are best used as preventative therapy, or as treatment for impaired cognitive function during active MDD episodes, or for residual cognitive impairments in remitted MDD.

Efforts to account for the heterogeneity of premorbid intelligence quotients between intervention groups have been made (e.g. [106]), making use of reliable [233] measures of reading and vocabulary, including the National Adult Reading Test; however, as Douglas et al. [234] note, these measures may not generalise to broad metrics of memory, learning and executive function used within studies. A potential solution is subgroup analysis of patient clusters based on cognitive presentations (e.g. working memory impaired vs. intact), although this requires large samples for sufficient power; alternatively, future studies could restrict study inclusion to participants with particular cognitive presentations that align with a priori hypotheses about the cognitive effects of the drug within MDD.

### Discrepancies in cognitive outcome measurement

There is considerable heterogeneity in how cognition is measured within the reviewed literature, with both objective neuropsychological measures and subjective self-report of the cognitive function used as outcome measures. Importantly, objective and self-report measures are not reliably correlated [235, 236]. While self-report measures may highlight cognitive deficits which are more meaningful to the individual with depression, they may be confounded by emotional state. Lower self-reported cognitive ability is known to be associated with higher depressive symptomatology [237], which may be the result of negative biases in appraisals of cognitive ability. Our consultations with individuals with lived experience of MDD highlighted the need for person-centred neuropsychological assessments, which focus on the specific cognitive impairments the individual describes affecting their lives. Given the benefits objective and subjective measures of cognition independently offer, both approaches should be used in future research investigating pro-cognitive efficacy of candidate compounds.

## Conclusions

Developing better treatments for cognitive impairment is an area of clinical priority in depression, underscored by accounts from individuals with lived experience of depression (Box 3). Throughout this review, we highlight pharmacological agents which hold promise (Table 1), including vortioxetine, modafinil and donepezil. These agents have complex mechanisms of action, and it is unclear whether cognitive change is mediated through primary neurotransmission or through indirect and/or intracellular processes. Better characterisation and consolidation of shared mechanisms between these agents may facilitate future drug discovery and development in this area [238]. There are novel agents such as fludrocortisone and erythropoietin which act on promising mechanistic targets beyond primary neurotransmission; the evidence is however preliminary, requiring further replication. Beyond this, the present review has highlighted multiple methodological challenges of human clinical research in this area (see Table 2), including pseudo-specificity and the selection of sensitive outcome measures.

**Table 1:** Pharmacological agents where improvements in cognitive function have been observed in individuals with MDD.

							Effect size of agent-related cognitive change^✝,^*		
Pharmacological agent	Agent properties	Study	Clinical population (*N*; age span)	Therapy type, dose	Periods of cognitive testing	Cognitive domain improved	Between-groups, post-intervention drug vs. placebo	Within-groups, repeated measures	Related affective change*
**Bupropion**	NA, DAT inhibition	[78]	MDD (*N* = 26; 20–50 yrs)	Monotherapy for 8 weeks, 150 mg q.d.	Week 0 and week 8	**↑** Verbal WM processing speed **↑** Attentional processing RT	*No placebo arm*	ε^2^ = 0.53 ε^2^ = 0.30	–
		[79]	MDD (*N* = 41; 20–50 yrs)	Monotherapy for 8 weeks, 150 mg q.d.	Week 0 and week 8	**↑** Immediate verbal recall **↑** Delayed verbal recall **↑** Immediate non-verbal recall **↑** Delayed non-verbal recall	*No placebo arm*	*g* = 0.44 *g* = 0.33 *g* = 0.91 *g* = 0.69	–
**Buspirone**	5-HT _1A_ partial agonist	[53]	MDD (*N* = 89, 20–65 yrs)	Adjunct with escitalopram for 8 weeks, starting at 5 mg b.i.d; titrated to 60 mg q.d	Week 0 and week 8	**↑** Digit span **↑ V**erbal fluency ^a^	*No placebo arm*	*g* = 0.81 *g* = 0.70	–
**DHEA (**+**Sulfate)**	Exogenous steroid/sulfate ester	[174]	MDD (*N* = 6, 51–72 yrs)	Monotherapy for 4 weeks, 30–90 mg q.d.	Week 0 and week 3	**↑** Verbal recall accuracy	*No placebo arm*	*g* = 0.84	–
**Donepezil**	Cholinesterase inhibitor; sigma-1 receptor agonist	[140]	MDD (*N* = 130, ≥65 yrs)	Adjunct to standard antidepressants for 2 years, 5 mg q.d. or 10 mg q.d.	Day 0; Week 52; Week 104	**↑** Global cognition ^b^	*d* = 0.27 (week 52); <0.05 (week 104)	–	–
		[141]	MDD (*N* = 21, ≥50 yrs)	Adjunct to antidepressants, starting at 5 mg q.d. for 12 or 52 weeeks, titrated to 10 mg q.d.	Week 8 (Baseline); Week 20; Week 52	**↑** Immediate verbal recall	*–*	*g* = 0.41 (week 8–20); 0.42 (week 8–52)	–
**Duloxetine**	NA, 5-HTT inhibition	[243]	MDD (*N* = 21, 18–45 yrs)	Monotherapy for 12 weeks, starting at 30 mg/day for first 4 days which increased to 60 mg/day	Week 0 and week 12	**↑** Psychomotor function ↑ Visual WM accuracy ↑ Visual WM processing speed ↑ Pattern recognition accuracy ↑ Immediate verbal recall accuracy ↑ Response inhibition	*No placebo arm*	*g* = 0.46 *g* = 1.30 *g* = 0.74 *g* = 0.46 *g* = 0.51 *g* = 0.61	Change in verbal WM related to symptomatic change
		[244]	MDD (*N* = 311, ≥65 yrs)	Monotherapy for 8 weeks, 60 mg q.d.	Week 0 and week 8	**↑** Verbal learning and memory	*Insufficient data*	*Insufficient data*	Related to reduced depressive symptoms
		[245]	MDD (*N* = 508, 18–65 yrs)	Monotherapy for 8 weeks, 10–20 mg q.d	Weeks 0 (Baseline) and 8	**↑** DSST performance **↑** Subjective executive functioning	*d* = 0.18 *–*	–	No relationship observed
**Erythropoietin**	Exogenous protein growth factor	[191]	TRD (*N* = 40, 18–65 yrs)	Adjunct with standard antidepressant for, 3 weekly 40000 IU/ml infusions for 8 weeks	Week 1 (Baseline); Week 9; Week 14	**↑** Verbal learning and memory recall composite	η_p_^2^ = 0.25 (week 1–9); 0.16 (week 14)	–	No relationship observed
		[192]	MDD and BD (*N* = 79, 40–49 yrs)	Adjunct with standard antidepressant, weekly 40,000 IU/ml infusions for 8 weeks	Week 1 (Baseline); Week 9; Week 14	**↑** Complex cognitive processing ^c^	η_p_^2^ = 0.13 (week 1–9); 0.08 (week 14)	–	–
**Fludrocortisone**	Mineralocorticoid receptor agonist	[187]	MDD (*n* = 24, 18–40 yrs)	Monotherapy for 5 days, 0.4 mg q.d.	1.5 hr after dose	**↑** Verbal memory **↑** Executive function	η_p_^2^ = 0.15 η_p_^2^ = 0.18	No repeated measures data	–
**Fluoxetine**	5-HTT inhibition	[181]	MDD (N = 40, 60–80 yrs)	Monotherapy for 6 weeks, fluoxetine 20 mg q.d. or mianserin 40 mg q.d.	Week 1 (Baseline); Week 6	**↑** Visuo-spatial working memory	*Insufficient data*	*Insufficient data*	–
**Org 26576**	Positive allosteric modulator of AMPA	[123]	MDD (*N* = 30, 18–65 yrs)	Monotherapy for 1 month, 100 mg BID or 400 mg BID	Days 1 (Baseline), 7, 14 and 28 (Post)	**↑** Executive functioning	*g* = 1.06 (100 mg BID); 0.65 (400 mg BID)	*–*	–
**Ketamine**	NMDA antagonist	[99]	MDD (*N* = 47, no data)	Adjunct with standard antidepressants and augmenting agents, single dose of 0.25 mg/kg S-ketamine or 0.5 mg/kg racemic ketamine	Day 1 (Baseline); Day 2 (Post)	**↑** Improved cognitive impairment depressive symptoms	*No placebo arm*	*g* = 0.60	–
		[109]	TRD (*N* = 71, 21–65 yrs)	Monotherapy, single dose 0.2-mg/kg or 0.5 mg/kg	Days 1 (Baseline), 3 and 14	**↑** Response control ^d^	*Insufficient data*	*Insufficient data*	–
		[105]	TRD (*N* = 43, 18–75 yrs)	Single 0.5 mg/kg IV and five 0.045 mg/kg midazolam IVs (placebo) or six 0.5 mg/kg IVs (intervention)	Day 1 (Baseline); Day 13	**↑** Processing speed **↑** Set-shifting ability **↑** Complex working memory **↑** Continuous visual recognition **↑** Visuospatial WM	η_p_^2^ = 0.14 η_p_^2^ = 0.09 – – η_p_^2^ = 0.08	η_p_^2^ = 0.21 η_p_^2^ = 0.34 η_p_^2^ = 0.27 η_p_^2^ = 0.20 *–*	No relationship observed
		[98]	TRD (*N* = 15, 18–70 yrs)	Adjunct with standard antidepressant and augmenting agents, 0.5 mg/kg 3 times weekly over a 12-day period	Weeks 1 (Baseline), 3, 4, 5 and 6	**↑** Continuous visual recognition Simple WM **↑** Complex WM	*No placebo arm*	η_p_^2^ = 0.38 η_p_^2^ = 0.35 η_p_^2^ = 0.34	Changes related to symptomatic change
		[107]	TRD with anxiety symptoms ^e^ (*N* = 30, 18–65 yrs)	Adjunct standard antidepressant and augmenting agents, six IVs of 0.5 mg/kg	Days 1 (Baseline), 13 and 26	**↑** Speed of processing **↑** Verbal learning and memory	*No placebo arm*	*g* = 0.93 (Day 13); 0.95 (Day 26) *g* = 0.51 (Day 13 only)	–
		[108]	TRD (*N* = 68, ≥18 yrs)	Adjunct with standard antidepressants, four infusions of 0.5–0.75 mg/kg IV	Day 0 (Baseline) and 7	**↑** DSST performance **↑** Attentional processing and task-switching **↑** General subjective cognitive function	*No placebo arm*	η_p_^2^ = 0.26 η_p_^2^ = 0.41 η_p_^2^ = 0.46	DSST and subjective cognition improvements mediated by symptomatic change
**Melatonin**	Exogeneous pineal hormone	[189]	MDD (*N* = 113, no data)	Adjunct with buspirone for 6 weeks, 15 mg q.d.	Weeks 1 (Baseline), 2, 4 and 6	**↑** Self-report cognitive functioning	–	η_p_^2^ = 0.603 ^f^	No relationship observed
**Memantine**	5-HTT inhibition	[119]	MDD with subjective memory complaints (*N* = 62, ≥60 years)	Adjunct with escitalopram for 1 year, mean daily dose of 11.1 mg q.d.	Week 0 (Baseline); Week 24; Week 52	**↑** Delayed verbal recall **↑** Executive function **↑** Global cognition	η_p_^2^ = 0.09 (Week 52) η_p_^2^ = 0.11 (Week 52) η_p_^2^ = 0.15 (Week 52)	–	No relationship observed
**Modafinil**	DAT inhibitor	[81]	MDD (*N* = 31, no data)	Adjunct with standard antidepressants for 4 weeks, starting at 100 mg q.d., titrated to 400 mg q.d.	Weeks 1 (Baseline), 2 and 4	**↑** Executive function	*Insufficient data*	*Insufficient data*	–
		[82]	Remitted MDD (*N* = 60, 18–65 yrs)	Adjunct with standard antidepressant for one day, 200 mg q.d.	Pre-dose; 2 hr after initial dose	**↑** Episodic memory **↑** Working memory	η_p_^2^ = 0.10 η_p_^2^ = 0.05	*–*	–
**Reboxetine**	NA inhibition	[204]	MDD (*N* = 74, 18–65 yrs)	Monotherapy for 8 weeks, 8–10 mg q.d.	Day 0 (Baseline), 14 and 56	**↑** Attentional processing **↑** Executive function	*Insufficient data*	*Insufficient data*	–
**SAMe**	Methyl co-factor	[201]	MDD (*N* = 55, 18–80 yrs)	Adjunct with standard antidepressant for 6 weeks, starting at 800 mg q.d., which increased to 1600mg q.d. after two weeks	Weeks 0 (Baseline) and 6	**↑** Subjective word recall	*g* = 0.42	–	–
**Tandospirone**	5-HT _1A_ agonist	[56]	Vascular MDD (N = 89, ≥50 yrs)	Adjunct with escitalopram for 8 weeks, 5 mg t.i.d	Weeks 0 (Baseline), 4 and 8	**↑** MMSE performance	*g* = 0.59 (Week 4); 1.31 (Week 8)	–	–
**Vortioxetine**	5-HTT inhibition; 5-HT _1A- B_ agonist_;_ 5-HT_3_, 5-HT_7_ and 5-HT_1D_ antagonist	[245]	MDD (*N* = 508, 18–65 yrs)	Monotherapy for 8 weeks, 10–20 mg q.d	Weeks 0 (Baseline) and 8	**↑** DSST performance **↑** Subjective executive functioning	*d* = 0.25 *Insufficient data*	–	No relationship observed
		[42]	MDD (*N* = 598, 18–65 yrs)	Monotherapy for 8 weeks, 10 mg or 20 mg q.d	Week 0 (Baseline), 1 and 8	**↑** DSST performance **↑** Verbal learning and memory **↑** Executive function **↑** Attentional processing **↑** Processing speed **↑** Subjective memory and executive functioning	*d* = 0.52 ^g^ *d* = 0.27 ^g^ *d* = 0.35 ^g^ *d* = 0.32 ^g^ *d* = 0.34 ^g^ *Insufficient data*	–	No relationship observed
**Venlafaxine**	NA, 5-HTT inhibition	[246]	MDD (*N* = 64, ≥18 yrs)	Monotherapy for 6 weeks, starting at 75 mg q.d. which gradually increased up to 75–150 mg daily	Weeks 1 (Baseline) and 6	**↑** Attentional processing error rate **↑** Attentional processing response time	*No placebo arm*	η_p_^2^ = 0.14 η_p_^2^ = 0.41	–

^a^Buspirone augmentation on cognitive improvement was only significant in individuals with MDD without atypical features.

^b^This effect size refers to a time x treatment effect for global cognition (processing speed, executive function, verbal memory, language, and visuospatial ability) and was only significant at week 52.

This increase was significant at 1 year although the effect size was small and not sustained at 2 years.

^c^Complex cognitive processing refers to a composite score across several cognitive measures, including tasks of verbal learning and memory, processing speed, simple and sustained attention, and executive functioning

^d^The significant slight increase in sustained attention and response control was only found with 0.5mg/kg of ketamine.

^e^Significant improvements in speed of processing and verbal learning and memory were only found in individuals with anxious TRD but not among individuals with non-anxious TRD.

^f^This effect size refers to treatment (melatonin adjunct to buspirone) non-responders only

^g^These effect sizes are averaged from cognitive outcomes for the vortioxetine 10mg q.d. and 20mg q.d. treatment groups.

*Cells containing “--” denotes analysis not undertaken or effect is not statistically significant.

^✝^Effect size is dependent on analysis performed: Hedges’ *g* (*g*) is reported for significant t-test analyses between intervention groups or across two time points (usually baseline and follow-up). Partial eta squared (η_p_^2^), Cohen’s *d* (*d*) or epsilon squared (ε^2^) is reported for analysis of variance models where a significant main effect of intervention group or across two time points was found. Hedges’ *g* or partial eta squared were derived from the study report where available or calculated where appropriate statistical values were reported. Values for *g*, *d* and η_p_^2^ were polarised to positive values where appropriate.

**Note:** Meta-analyses discussed within the review were not included in the above table; only single studies where positive cognitive properties were observed in a psychiatric population, predominantly MDD or TRD, are included.

*DHEA(-S)* refers to dehydroepiandrosterone steroid precursor or sulfate forms, *SAMe* factor S-Adenosylmethionine, *MMSE* Mini-mental state examination, *DSST* Digit symbol substitution test. *WM* Working Memory, *RT* Response Time.

**Table 2:** Key considerations for investigations of pharmacological interventions which target cognitive impairment in depression.

**Pseudo-specificity**	*Description:*	Indirect gains in cognitive performance due to changes in affective processing or motivation.
	*Considerations:*	• Statistical factor analysis to determine that cognitive change is independent of affective gain. • Use of relevant measures of affect and motivation, not only symptom-based outcome measures (e.g. BDI, MADRS).
**Consistency of translation**	*Description:*	Translation of cognitive effect between healthy and depressed populations is not always observed.
	*Considerations:*	• Establishing translational biomarker models of cognitive treatment response. • Use of cognitive outcome measures which have greater sensitivity to cognitive change in healthy populations.
**Heterogeneity of cognitive impairment**	*Description:*	Cognitive impairment manifests differently for every individual with MDD, which has implications for the therapeutic utility of drugs which target specific domains of cognition.
	*Considerations:*	• Consider subgroup analysis of patient clusters based on cognitive presentation if samples provide adequate power. • Consider recruitment of samples with specific cognitive presentation.
**Discrepancies in cognitive measurement**	*Description:*	Non-uniform approach to cognitive domains assessed across research studies, particularly regarding the differential use of objective and subjective measures of cognition.
	*Considerations:*	• Adopt a uniform approach to measurement; standardised battery of tasks appropriate for the heterogeneous profile and functional consequences of cognitive impairment in MDD. • Consider a combination of both objective and subjective cognitive outcome measures.
**Therapeutic specificity**	*Description:*	Conditions necessary for successful clinical use of agents which target cognition. In particular, whether efficacy is only seen in individuals who are currently depressed, or also in those at risk of depression and/or with remitted depression.
	*Considerations:*	• Identify whether agents should be used as prevention or treatment. • Identify whether treatment should be given during active episodes or to treat residual impairments during remission.

While the present review is limited in scope to MDD, cognitive deficits in neuropsychiatric populations may be considered transdiagnostically [239], and as such it is worth considering the generalisability of clinical pharmacological evidence beyond MDD. In addition, this review has focussed on non-affective cognitive function, although there are many aspects of affective cognition that are known to play a core role role in depression, such as rumination and emotion regulation [240]. Given the association between impaired executive functioning and increased rumination [241] and reduced cognitive reappraisals [242], future research may consider if these aspects of psychopathology are influenced by drugs which improve cognitive function in depression.

Beyond those reviewed here, there are many further novel promising targets for improving cognitive impairment in MDD where placebo-controlled RCTs have yet to demonstrate cognitive improvement in MDD, or the agents have not yet crossed the preclinical threshold, including creatine, α2-adrenergic receptor antagonists, glucagon-like peptide-1 agonists, GABA_B_ receptor agonists, 5-HT_1A_ biased agonists, and histamine H_3_ receptor antagonists. With continued efforts in this space, and by adopting robust and consistent methodological approaches across the translational pipeline, there is real promise that the treatment of cognitive impairment in depression may be improved in the future.

Box 3 Openness to pharmacological approaches to improving cognitive impairment– accounts from individuals with lived experience of depression Throughout consultations, lived experience group members agreed they would be open to these interventions, both separately and in tandem, as appropriate.

- *“I would be open to a drug that enhances my cognitive ability, but I would want something with minimal side effects.”*
- *“I’m open to medication […], as long as it was specifically targeted and individualised for me.”*
- *“Targeting concentration would be a really good first step, because it would clear the way to targeting other things.”*

Finally, the lived experience group expressed a preference for treatments to be personalised, designed to consider the different ways in which depression and cognitive impairment may present in the individual.

- *“It needs to be patient-centred; you need to take them seriously, as they are the only one who knows what’s going through their brain.”*
- *“I felt that the team who had worked with me hadn’t treated me as an individual.”*

## Supplementary information

Supplementary Materials
