# Supplementary material for: Pharmacological targeting of cognitive impairment in depression: recent developments and challenges in human clinical research
Source: Transl Psychiatry. 2022 Nov 17;12:484. doi: 10.1038/s41398-022-02249-6 (PMC9671959; doi:10.1038/s41398-022-02249-6)
Supplement: Supplementary file 1 — Supplementary Materials [file 41398_2022_2249_MOESM1_ESM.docx]

**Supplementary Material 1 – Questions for individuals with lived experience of depression during PPI (Public and Patient Involvement) consultations (group and individual)**

- 1. “Are problems with cognition something you ever experienced at any point in your life? So for example, were there things you had difficulty doing “Did you have difficulty … forgetting where you put something, forgetting the word for something, difficulty concentrating, *etc”*

**Follow-up (after everyone has responded)** -- “When you were feeling low, did you also experience these problems, and were they any better or perhaps worse?

**Potential follow-up** – “How do you think this impacted your recovery?”

**Potential follow-up** – “Did it impact … your ability to work, interact with others, do the things you usually enjoy doing?”

**Potential follow**-**up** – “Do you feel that the inability to think or remember made depressed feelings worse?”

- 1. “When you were in services, was cognitive functioning (not cognitions – make clear!) something your healthcare supervisor talked about with you, or considered?

**Follow up –** “What kinds of things do you think they should have done / how did they consider it (*e.g.* assessment?)

- 1. “If your psychiatrist suggested a medication or a psychological intervention (for example something that would entail mental effort by repeating a language or math problems repeatedly with the help of a psychologist) to address cognitive problems – would you have been open to this?”

**Follow-up –** “Would you prefer medication or a psychological intervention?”

**Potential follow-up** – “Which do you think would require more motivation or be easier for you when you were feeling low?”

- 1. “Now we’ve discussed all of that – What do you think researchers should focus on the most, that would have helped best with cognitive impairment during your recovery? – It could be the things we’ve alluded to, like medication, or perhaps it’s something different?”

**Supplementary Material 2 – Literature search strategy: queried strings and combinations input into literature databases**

| **String category** | **Queried strings** |
| --- | --- |
| **#1 – Study Population** | “Depression” OR “Depressive Disorder” OR “Treatment-resistant depression” OR “Refractory depression” OR “Healthy Individuals” OR “Healthy Volunteers” OR “Older Adults” |
| **#2 – Study Methodology** | “Drug Study” OR “Clinical Study” OR “RCT” OR “Randomised Controlled Trial” OR “Meta Analysis” OR “Systematic Review” |
| **#3 – Cognitive State, Assessment or Domain** | “Cognitive Impairment” OR “Cognitive Functioning” OR “Cognitive Outcome” OR “Neuropsychological assessment” OR “Executive Functioning” OR “Decision making” OR “Long-term Memory” OR “Working Memory” OR “Metacognition” OR “Attention” OR “Psychomotor functioning” OR “Global Cognition” |
| **#4 – Neurochemical Targets (prefix)** | “Dopamine*” OR “Norepinephrine” OR “Noradrenaline” OR “Serotonin*” OR “5-HT*” OR “Adrenergic” OR “Acetylcholine” OR “Cholinergic” OR “Cholinesterase” OR “GABA*” OR “Adrenaline” OR “Epinephrine” OR “Glutamate*” OR “NMDA” OR “AMPA” OR “Sigma*” OR “Hormone” OR “*steroid” OR “Dehydroepiandrosterone” OR “DHEA” OR “Progesterone” OR “Cortico*” OR “Histamine*” OR “Creatine” OR “Monoamine” OR “Neuropeptide” OR “BDNF” OR “Growth Factor” OR “Insulin” |
| **#5 – Neurochemical Targets (supplementary string)** | “Receptor” OR “Transporter” OR “Metabotropic” OR “Exogenous” OR “Neuroinflammatory” OR “Signalling” |
| **#6 – Neurochemical Mechanism (supplementary string)** | “Agonist” OR “Biased Agonist” OR “Antagonist” OR “Biased Antagonist” OR “Inhibitor” OR “Modulator” OR “Multimodal” |
| **#7 – Agent Class or Name** | “SSRI” OR “SNRI” OR “Antidepressant*” OR “Nootropic*” OR “Stimulant*” OR “Bupropion” OR “Buspirone” OR “Donepezil” OR “Duloxetine” OR “Erythropoietin” OR “*citalopram” OR “Fludrocortisone” OR “Fluvoxamine” OR “Framptor” OR “*ketamine” OR “Melatonin” OR “Modafinil” OR “Reboxetine” OR “S-Adenosylmethionine” OR “Tandospirone” OR “Vortioxetine” OR “Venlafaxine” |
| **String query combinations:** | *To isolate human pharmacological studies or systematic reviews based on neurochemical targets:*  String category #1 AND #2 AND #3 AND #4 AND #5  String category #1 AND #2 AND #3 AND #4 AND #6  String category #1 AND #2 AND #3 AND #4 AND #5 AND #6  *To isolate human pharmacological studies or systematic reviews based on known agent classes or names:*  String category #1 AND #3 AND #7  String category #1 AND #2 AND #3 AND #7 |

**Note:** Multiple strings may have been used per string category within respective combinations. Strings were queried across the following databases: PubMed, Web of Science and Google Scholar. The asterisk (*) wildcard queries any potential prefixes or suffixes for each string.
